# Topical oxybutynin antiperspirant for axillary hyperhidrosis: a phase IIb PROBE design clinical trial (TODAY trial)

**DOI:** 10.1590/1677-5449.20250065

**Published:** 2026-09-14

**Authors:** Samantha Neves, Elisete Isabel Crocco, Viviane Santana da Silva, Felipe Menegueti, Alexia Paganotti, Ricardo Sanchez Boix, Nelson Wolosker, Roberto Augusto Caffaro

**Affiliations:** 1 Irmandade da Santa Casa de Misericórdia de São Paulo, São Paulo, SP, Brasil.; 2 Science Valley Research Institute, Santo André, SP, Brasil.; 3 Faculdade de Medicina do ABC - FMABC, Santo André, SP, Brasil.; 4 Hospital Israelita Albert Einstein, São Paulo, SP, Brasil.; 5 Hospital Alemão Oswaldo Cruz, São Paulo, SP, Brasil.

**Keywords:** dermatology, axillary hyperhidrosis, antiperspirant, oxybutynin, dermatologia, hiperidrose axilar, antiperspirante, oxibutinina

## Abstract

**Background:**

Primary hyperhidrosis is a chronic condition with limited therapeutic options.

**Objectives:**

To evaluate the impact of 10% oxybutynin spray on axillary hyperhidrosis, considering both its local and systemic effects, in terms of Hyperhidrosis Disease Severity Scale (HDSS) outcomes for hyperhidrosis of the axilla and eight additional sites.

**Methods:**

Prospective phase II PROBE design trial. Adult patients with axillary hyperhidrosis were randomized to receive a 10% oxybutynin topical spray, oral oxybutynin, or placebo spray for 35 days. Efficacy was evaluated on day 35 using the HDSS and a hyperhidrosis-specific quality-of-life (QoL) assessment questionnaire.

**Results:**

17 patients were randomized. No significant differences were observed in HDSS outcomes from baseline to day 35 in any of the three groups (2.5 [2 - 4], 4 [3 - 4], 3.5 [3 - 4], p= 0.207), although a trend to improvement was noted in both treatment groups. Significant improvement in the QoL questionnaire was observed from baseline to day 35 in the oxybutynin spray group (pre-intervention QoL: 78.5 [70 - 96] and post-intervention QoL: 38.5 [32 - 67], p = 0.031).

**Conclusions:**

No differences were observed in the primary outcome. A significant improvement from baseline to day 35 was observed in the QoL questionnaire score for the oxybutynin spray group.

## Resumo

**Contexto:**

A hiperidrose primária é uma condição crônica com opções terapêuticas limitadas.

**Objetivos:**

Avaliar o impacto do *spray* de oxibutinina a 10% na hiperidrose axilar, considerando seus efeitos local e sistêmico, em termos de desfechos da Escala de Gravidade da Doença de Hiperidrose (HDSS) para hiperidrose da axila e oito locais anatômicos adicionais.

**Métodos:**

Estudo prospectivo de fase II com desenho PROBE. Pacientes adultos com hiperidrose axilar foram randomizados para receber *spray* tópico de oxibutinina a 10%, oxibutinina oral ou *spray* placebo por 35 dias. A eficácia foi avaliada no dia 35 pela HDSS e por um questionário de avaliação da qualidade de vida (QV) específico para hiperidrose.

**Resultados:**

17 pacientes foram randomizados. Não foram observadas diferenças significativas nos resultados da HDSS desde o início até o dia 35 em qualquer um dos três grupos (2,5 [2 - 4], 4 [3 - 4], 3,5 [3 - 4], p=0,207), embora tenha sido observada tendência de melhora nos grupos tratados. Foi observada melhora significativa no questionário de QV do início até o dia 35 no grupo do *spray* de oxibutinina (QV pré-intervenção 78,5 [70 - 96] e QV pós-intervenção 38,5 [32 - 67], p = 0,031).

**Conclusões:**

Não foram observadas diferenças no desfecho primário. Foi observada melhora significativa no questionário de QV do início até o dia 35 no grupo do *spray* de oxibutinina.

## INTRODUCTION

Hyperhidrosis (HH) is a condition characterized by excessive sweating that goes beyond what is necessary to regulate body temperature.^1^ It affects approximately 15.3 million individuals in North America.^2^ The etiology of primary HH remains poorly defined, but is thought to involve overactivity of the sympathetic nervous system.^3^ HH has a significant impact on quality of life (QoL) and is associated with social and professional stigma, similar to chronic conditions.^4,5^ Primary HH (PHH) accounts for 90% of HH cases.^6-8^

Current treatment options for PHH include video-assisted thoracoscopic sympathectomy (VATS), botulinum toxin injections, various topical therapies, and oral anticholinergics. However, these treatments vary in effectiveness and can cause significant side effects.^9-11,12^ Transdermal delivery of anticholinergics reduces hepatic metabolism, thereby decreasing systemic exposure and the incidence of adverse effects, such as xerostomia.^13^

There are limited reports on topical anticholinergics, but topical glycopyrrolate in cloth and cream form and oxybutynin in gel form are both effective.^14-17^ The oxybutynin doses reported in the literature for hyperhidrosis range from 1% to 20%, and the vehicles used include lotion and gel.^18,19,20,21,22^ Topical oxybutynin spray for axillary HH is easier to use, but has not been tested previously. We hypothesized that topical oxybutynin spray would be non-inferior to oral oxybutynin for treating axillary HH, at the dose already established in the literature.^11^ The hypothesized benefit of a 10% oxybutynin spray for axillary HH lies in its limited systemic effects, which may reduce the sympathomimetic side effects seen with oral anticholinergics and potentially improve treatment adherence and improve both hyperhidrosis-specific QoL questionnaire (QoLQ) and Hyperhidrosis Disease Severity Scale (HDSS) scores.^23,24^

This phase II exploratory study aimed to evaluate the efficacy of a 10% oxybutynin spray, applied twice daily, compared to oral oxybutynin and topical placebo in patients with axillary HH, using HDSS scores to assess efficacy.

## METHODS

### Study design and oversight

TODAY was an academically led, investigator-initiated, pragmatic, multicenter, phase IIb hypothesis-generating randomized clinical trial. The study was open label with blinded endpoint assessment [a prospective randomized open blinded endpoint (PROBE) study]. It was conducted at the private office of the principal investigator and at the outpatient hyperidrosis clinic at the Faculty of Medical Sciences of the Santa Casa de Misericórdia de São Paulo, São Paulo, Brazil. Patients with axillary HH were randomized and assigned into one of three groups. Group A received a 10% oxybutynin spray, two sprays per axilla twice a day for 35 days. Group B received oral oxybutynin for 35 days. Group C received a topical placebo applying two sprays per axilla twice a day for 35 days. Patients received pre-screening and a 35-day post-treatment questionnaire, the HDSS, and a QoLQ (Figure 1). Randomization was balanced through variable block sizes to ensure allocation concealment, using the RedCap platform. The randomization sequence was generated before study initiation and assignment occurred at the time of enrollment through the secure electronic platform. The study employed an open-label design. Details of the study design have been reported previously.^25^

**Figure 1:**
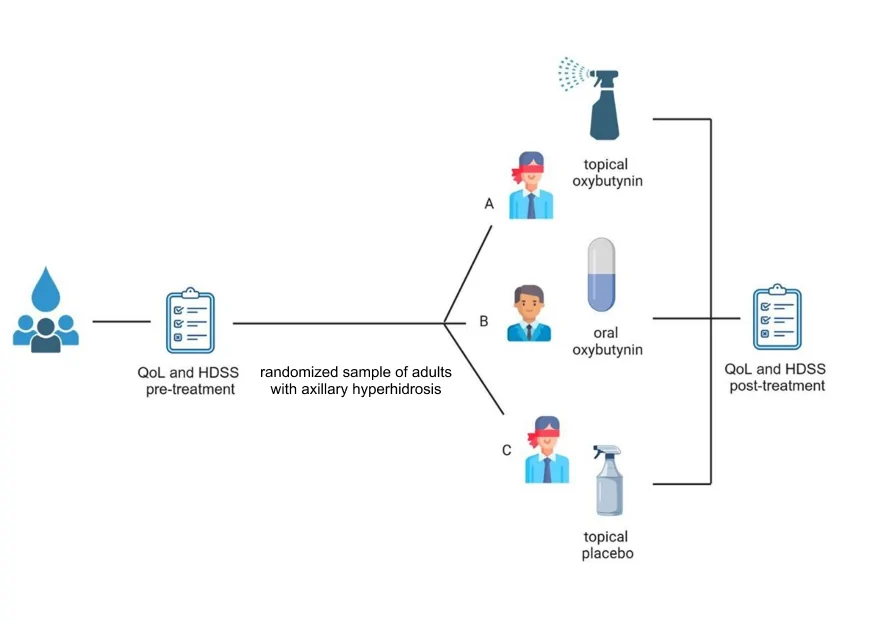
Study design. (A), (B), (C): three study arms.

The trial was conducted at two centers in Brazil from September 2020 to June 2024. The trial protocol has been published previously and was approved by the institutional review boards at all trial sites (Approval number: 3.978.439/CAAE: 65651422.6.0000.9147). The first and senior authors designed the trial. The first draft of the manuscript was written by the first author and revised based on comments from all the authors. No writing assistance was provided. The trial was registered at ClinicalTrials.gov, identifier: NCT05102396.

### Inclusion and exclusion criteria

Eligible patients were between 18 and 45 years of age. Patients with axillary HH were diagnosed by a dermatologist, based on at least four of the following medical history criteria: a. HH in the armpits; b. bilateral and symmetrical symptoms; c. sweating that impairs daily activities; d. at least weekly occurrence; e. onset < 25 years; f. positive family history; and/or g. cessation during sleep. All patients had an average hyperhidrosis-specific QoLQ score greater than 69.

Exclusion criteria included patients who are hypersensitive to oral oxybutynin hydrochloride; patients diagnosed with systemic diseases or dermatological diseases in the armpit, such as psoriasis or intertrigo; patients with angle-closure glaucoma, total and partial obstruction of the intestinal tract, ulcerative colitis, megacolon, myasthenia gravis, or heart disease; patients who were pregnant or intending to become pregnant and patients with symptoms of menopause; and patients with signs of skin lesions in the armpit.

### Trial procedures

After screening, patients signed informed consent and underwent standard clinical evaluations conducted by a trained dermatologist. Data were collected on baseline characteristics, including demographics, comorbid conditions, and clinical history, using the RedCap^©^ software. Patients had an initial screening assessment, followed by administration of two questionnaires: the HDSS and QoLQ. Participants were randomly assigned in a 1:1:1 ratio to receive 10% oxybutynin spray, oral oxybutynin, or topical placebo using a computer-generated randomization sequence (RedCap^©^). They were instructed to avoid treatments that could affect sweating (toxin botulinum, oral anticholinergics or topical anticholinergics, and topical aluminum chloride at concentrations exceeding 19%). Patients using regular deodorants were permitted to continue daily use. Patients were encouraged to comply with the treatment and the final two questionnaires were administered on day 35 (Figure 1).

The TODAY trial products were supplied by Pharmedica compounding pharmacy. Oral oxybutynin is currently approved by ANVISA (Brazilian regulatory agency) for treatment of patients with urinary incontinence. Its use for HH is off label. The 10% oxybutynin spray (investigational product) used in group A was used twice a day, administering two sprays per axilla. The composition of topical oxybutynin was 10% oxybutynin hydrochloride, 30% grain alcohol and enough water to make up a 60 ml spray. Each 10% oxybutynin spray administered 0.15 ml containing 0.015 grams or 15 milligrams of oxybutynin. As 2 sprays were applied to each armpit, the dose delivered per application per armpit was 30 milligrams of oxybutynin. The patients were not instructed on whether to shave their armpits.

The same dose of oral oxybutynin as used for urinary incontinence was used in group B, (capsules containing 2.5 mg of oral oxybutynin once daily, at night, for the first seven days, capsules containing 2.5 mg twice a day from the 8th to the 21st day, and capsules containing 5 mg twice daily from the 22nd to the 35th day). The spray used in group C was a topical placebo mimicking the topical oxybutynin spray and was made using an identical alcoholic vehicle.

### Hyperhidrosis Disease Scale Severity

The HDSS was developed by the International Hyperhidrosis Society^®^ and is designed to be a rapid assessment tool comprising a single question with four answers. The HDSS is simple, facilitating ease of response and minimizing the likelihood of errors. A validated Portuguese version of the HDSS was used.^24^ Grade 1 indicates no perceptible sweating or interference in daily life; grade 2 is tolerable sweating with occasional interference; grade 3 is moderately tolerable sweating with frequent interference; and grade 4 is intolerable sweating with constant interference. Patients were asked to assign a specific HDSS score to each of the nine anatomical sites commonly affected by hyperhidrosis, including the axillae. For data analysis, an improvement delta was calculated based on HDSS scores, defined as the difference between the HDSS score at baseline (week 0) and the HDSS score at the follow-up visit. A delta greater than or equal to 1 was considered indicative of improvement.

### Quality of Life Questionnaire

Each patient completed a QoLQ. This is a validated Portuguese questionnaire^23^ with a maximum score of 100 points, with higher scores indicating worse QoL. The negative impact of hyperhidrosis on QoL before treatment was classified into five distinct satisfaction levels, calculated based on total QoL score (ranging from 20 to 100). A score greater than or equal to 84 indicates very poor QoL; 68 to 83 indicates poor QoL; 52 to 67 indicates good QoL; 36 to 51 indicates very good QoL; and 20 to 35 indicates excellent QoL. After treatment, the absolute QoL score was re-evaluated using the same five distinct levels**.**.

### Outcomes

#### Primary outcome

The primary efficacy outcome was improvement in axillary HH severity at 35 days, as measured by the HDSS. A treatment responder was defined as any participant whose initial HDSS score was 3 or 4 and who achieved at least a 1-point improvement in the scale by day 35. Additionally, the impact on quality of life was evaluated separately by comparing the absolute QoLQ scores between baseline and day 35

#### Secondary outcomes

Secondary efficacy outcomes included improvement in HH severity at the other 8 sites at 35 days, as measured by the HDSS.

The secondary safety outcome was clinical assessment of adverse events, including dry mouth and skin damage.

### Statistical analysis

Friedman tests were used to evaluate the effect of assessment time (pre/post) on QoLQ and HDSS scores by anatomical site for each study group (topical/oral/placebo). Interaction analyses between the factors “time” and “group” could not be conducted because of the small sample size and the characteristics of the study variables (ordinal). For the same reason, it was also impossible to estimate the significance of the proportion improvement in HDSS scores. Statistical analyses were conducted and graphs were plotted using R (RStudio: Integrated Development for R. RStudio, PBC, Boston, 2020), adopting a 5% significance level (α = 0.05).

For the secondary efficacy endpoint, side effects were evaluated descriptively, utilizing percentage frequencies to facilitate comparison between groups (since they have different sample sizes).

Mean ± standard deviation was used for the age assessment. A Kruskal-Wallis analysis was used to compare the ages of different groups. Side effects were evaluated descriptively using percentage frequencies to allow comparison between groups (since they have different sample sizes). Given that this is a hypothesis generating phase IIb study, no sample size calculations were performed. The sample for this study was a convenience sample, based on previous phase II b studies. This study is sized to provide data for future, more precise, sample size calculations.

## RESULTS

From September 2022 to June 2024, seventeen adult patients with axillary HH were randomized into three groups at 2 centers in São Paulo, Brazil. The mean age was 27.6 years (standard deviation of 9.1 years). Ten participants were female (71.43%), seven were black, and seven were white, reflecting diversity. Baseline characteristics are shown in Table 1. Fourteen patients completed the 35-day treatment period. One patient randomized to the oral oxybutynin group missed a follow-up visit and one patient randomized to oral oxybutynin withdrew informed consent due to adverse effects (Figure 2).

**Table 1:** Baseline characteristics.

	**Oxybutynin Spray**	**Oral Oxybutynin**	**Placebo Spray**
Female sex – n (%)	5 (83.33)	2 (50)	3 (75)
Age, median (range) - Years	30.5 (19-43)	20.3 (18-27)	30.8 (18-44)
Race – n (%)			
White	3 (50)	2 (50)	2 (50)
Black	3 (50)	2 (50)	2 (50)
Other	-	-	-

**Figure 2:**
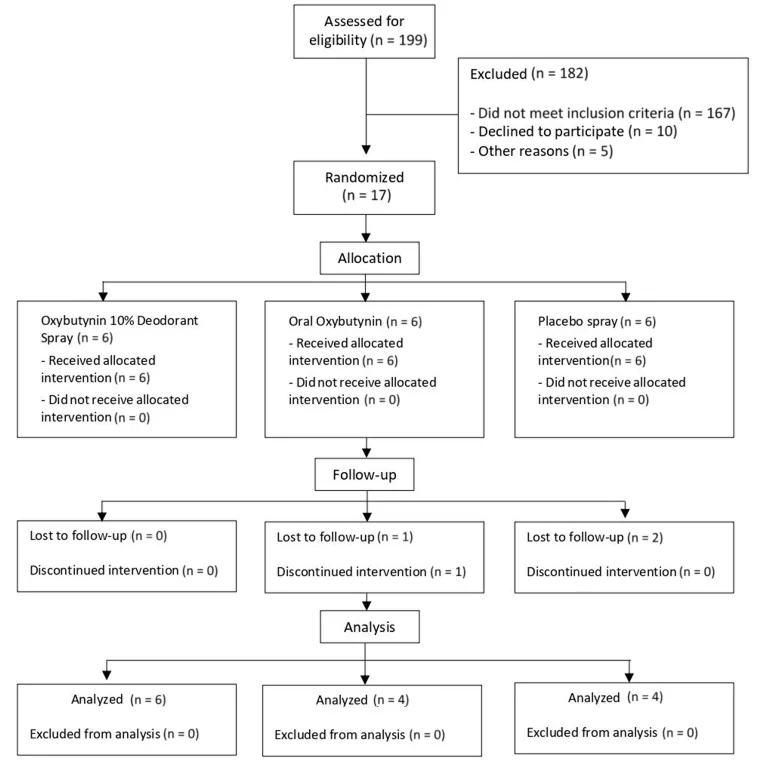
TODAY trial CONSORT diagram.

### Improvement in HDSS scores

Four patients treated with 10% oxybutynin spray had an axillary HH improvement of at least 1 point. Similarly, four patients on oral oxybutynin treatment also had an axillary HH improvement of at least 1 point Additionally, four patients in the placebo group did not show any improvement in axillary HH.

No significant differences were observed in the 10% oxybutynin spray group versus oral oxybutynin group, either between different assessment times (within the same group) or between different groups (at the same assessment time) (Table 2).

**Table 2:** Primary efficacy results according to the HDSS questionnaire, by anatomical area.

**Variable**	**Time**	**TOPICAL (n=6)**	**ORAL (n=4)**	**PLACEBO (n=4)**	**p** *
HDSS Axillae	PRE	2.5 (2 - 4)	4 (3 - 4)	3.5 (3 - 4)	0.207
	POST	2 (1 - 3)	2 (1 - 3)	3.5 (2 - 4)	0.160
	p**	0.313	0.125	>0.999	
HDSS Hands	PRE	2 (1 - 4)	2.5 (1 - 4)	3 (1 - 4)	0.691
	POST	1.5 (1 - 2)	1.5 (1 - 3)	2.5 (1 - 4)	0.409
	p**	0.500	0.500	>0.999	
HDSS Head	PRE	2.5 (1 - 4)	2 (1 - 2)	2.5 (2 - 4)	0.317
	POST	1 (1 - 3)	1 (1 - 2)	2 (1 - 4)	0.385
	p**	0.063	0.500	0.500	
HDSS Feet	PRE	2 (2 - 4)	3.5 (1 - 4)	2.5 (1 - 4)	0.853
	POST	2 (1 - 4)	2 (1 - 2)	2.5 (1 - 4)	0.928
	p**	0.5	0.375	-	
HDSS Dorsum	PRE	1.5 (1 - 4)	2.5 (2 - 4)	2.5 (2 - 3)	0.259
	POST	1.5 (1 - 3)	1.5 (1 - 3)	2 (2 - 4)	0.344
	p**	>0.999	0.250	>0.999	
HDSS Abdomen	PRE	1.5 (1 - 3)	2.5 (1 - 3)	1.5 (1 - 3)	0.652
	POST	1.5 (1 - 3)	1 (1 - 2)	2 (1 - 3)	0.379
	p**	>0.999	0.250	>0.999	
HDSS Thorax	PRE	1 (1 - 3)	3 (1 - 4)	2 (2 - 3)	0.166
	POST	2 (1 - 2)	1.5 (1 - 2)	2 (1 - 3)	0.659
	p**	>0.999	0.250	>0.999	
HDSS Groin	PRE	2 (1 - 2)	1.5 (1 - 2)	2.5 (1 - 3)	0.395
	POST	1 (1 - 3)	1 (1 - 2)	2.5 (1 - 4)	0.278
	p**	>0.999	>0.999	>0.999	
HDSS Buttocks	PRE	1 (1 - 3)	2 (2 - 4)	2.5 (1 - 3)	0.225
	POST	1.5 (1 - 3)	1 (1 - 3)	2 (1 - 4)	0.586
	p**	>0.999	0.125	>0.999	

* p value according to the Kruskal-Wallis test between different groups (within the same time).

** p value according to the Wilcoxon test between different assessment times (within the same group).

Comparison of the anatomical positions with the highest proportions of improvement showed that the axilla and foot were the areas with the highest proportion of improvement in the oral oxybutynin group (100% each), the chest and head improved most in the placebo group (50% each), and the head (85%) and axilla (66%) exhibited greatest improvement in the 10% oxybutynin spray group.

### Improvement in QoL Questionnaire scores

The median scores of the QoL scales differed between the pre-intervention and post-intervention assessments for all three groups (with consistently better values post-intervention). However, no significant differences were observed between the groups (Table 3).

**Table 3:** Primary efficacy results for QoL questionnaire.

**Time**	**TOPICAL (n=6)**	**ORAL (n=4)**	**PLACEBO (n=4)**	**p** *
PRE	78.5 (70 - 96)	78.5 (70 - 86)	77.5 (71 - 92)	0.929
POST	38.5 (32 - 67)	41 (25 - 67)	59 (58 - 62)	0.246
p**	0.031	0.125	0.250	

* p value according to the Kruskal-Wallis test between different groups (within the same time).

** p value according to the Wilcoxon test between different assessment times (within the same group).

### Safety outcomes

All groups experienced side effects at some point. However, the placebo group had the lowest proportion of patients with side effects. On day 35, no patients on the placebo arm reported side effects (Figure 3).

**Figure 3:**
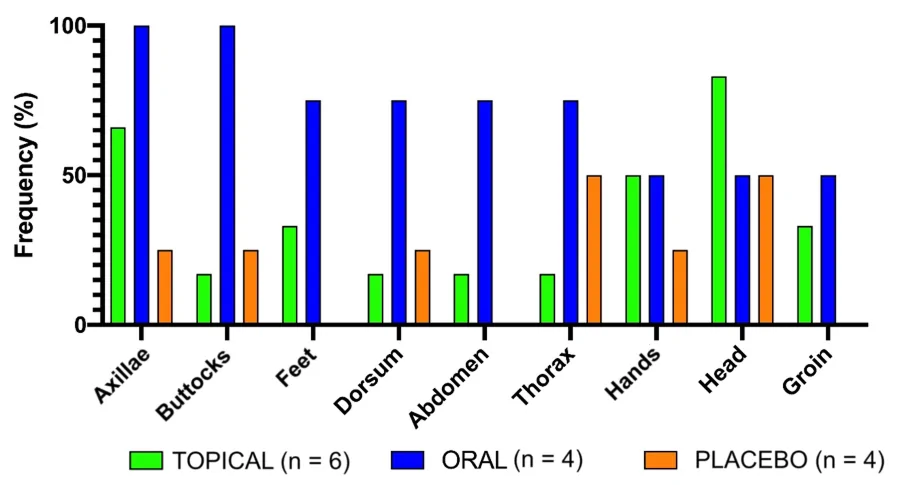
Bar graph showing differences in HDSS scores for hyperhidrosis, by anatomical regions, in all groups.

Two of the 6 patients in the 10% oxybutynin spray group had axillary irritation. All patients had xerostomia. Other side effects, such as constipation, urinary retention, and headaches, were each reported by more than two patients across the different study groups. All patients in the topical placebo group experienced side effects such as xerostomia, urinary retention, dizziness, and nausea. One of the four patients who completed the study had axillary burning sensation.

One out of the 17 patients discontinued treatment due to significant axillary irritation. Side effects were observed in all groups at some point. The topical placebo group had the lowest proportions of patients with systemic side effects.

All adverse events were mild, except for axillary irritation in one patient, which resulted in discontinuation of treatment. All adverse events resolved after withdrawal of treatment.

## DISCUSSION

The severity and location of primary HH can affect daily activities and professional careers. Studies have shown that patients typically report poor or very poor QoL before treatment, since those with better QoL do not usually seek treatment. The main goal of treating primary HH is to reduce sweat production, which leads to improved QoL by alleviating the psychological burden.^26,27^ This improvement occurs despite potential side effects, which are generally deemed insignificant by patients. In addition to impacting QoL, primary HH may also cause psychological disturbances and impair social relations.^28^

Devices that measure sweat evaporation from the skin or sweat can be used to quantitatively assess sweating. However, these instruments may not accurately reflect the daily distress experienced by individuals with HH. Therefore, patient-reported outcomes are crucial for evaluating the impact of HH. The HDSS and QoLQ assess primary HH progression subjectively. QoL assessments are essential tools in medical research, as they enable high-quality data collection for diagnosing and monitoring patient outcomes.^26^

Our study included a diverse population, with a sample comprising 64% women and including a significant number of black patients.

No statistically significant differences were observed in HDSS scores from baseline to day 35 in any of the 3 groups, which may be due to the small sample size (n=17) in this phase II exploratory study. The HDSS is the most sensitive patient-centered outcome for evaluating the impact of primary HH treatment and while the results did not attain statistical significance, they did reveal a clear trend to improvement in both therapeutic arms of the study (10% oxybutynin spray and oral oxybutynin) in comparison to placebo, particularly in the axillary region. Another way to assess the results is by observing the proportion of improvement on the HDSS scale after the intervention. It was observed that, with the exception of results for the head, results for all other areas showed a higher proportion of patients with improvement than without. Furthermore, this suggests there was a systemic effect in the 10% oxybutynin spray group, since, not only did their axillary results show improvement, but all areas assessed also did so, with at least 50% of patients.

The QoLQ results showed that 82.3% of patients had a score equal to or above 68. This means that 82.3% of patients with axillary HH had poor or very poor QoL. To further restrict the population, we suggest adopting QoLQ ≥ 68 as an inclusion criterion to make the post-treatment QoLQ results more accurate. Regardless, 83.33% of the patients allocated to the 10% oxybutynin spray group showed an improvement in QoL from baseline to day 35 (p=0.03). There was a significant improvement in QoLQ scores from baseline to day 35 in the 10% oxybutynin spray group. The lack of significance between the pre-intervention and post-intervention assessments in the other groups may be attributable to the small sample size. In these groups, QoL scores remained stable compared to baseline, indicating that the occurrence of side effects did not negatively impact their overall QoL. Side effects were observed in all groups at some point. The topical placebo group had the lowest proportions of patients with systemic side effects, suggesting an apparent systemic effect in the 10% oxybutynin spray group, similar to those observed in patients allocated to the oral oxybutynin group. The observation of systemic effects following topical oxybutynin administration does not support the premise that topical delivery precludes systemic side effects. On the other hand, the presence of these systemic effects may offer a collateral benefit by reducing sweating in areas beyond the initial site of administration. The axillary irritation observed in two of the six participants in the topical oxybutynin group suggests that the vehicle may be excessively irritating to the skin. Patients with hyperhidrosis typically maintain a rigorous axillary cleansing routine, which may have predisposed the skin to sensitization by the alcoholic vehicle rather than by the active pharmacological agent itself.

### Study strengths/limitations and future directions

This study was the first to investigate the impact of 10% oxybutynin spray in antiperspirant formulation on HDSS and hyperhidrosis-specific QoLQ scores for HH. The study was an open label design with blinded endpoint assessment (PROBE), decreasing the bias against using the double-blind design. Limitations include the small sample size for this phase II hypothesis-generating study, caused by the COVID-19 pandemic, which restricted patient recruitment and patient access to axillary HH diagnosis. Future studies will recruit larger patient groups treated with different concentrations of topical oxybutynin in antiperspirant formulations for extended periods (probably lower concentrations to reduce side effects). Such lower concentrations could support the benefit of combining topical and oral administration, thereby reducing undesirable systemic side effects while providing additional improvement in sweating in areas beyond the application site. Tests to detect oxybutynin in the blood are also planned to understand long-term outcomes.

## CONCLUSION

No significant differences were observed in the HDSS scores from baseline to day 35 in any of the 3 groups. However, there was a clear trend to improvement in scores for both questionnaires for both treatment groups (10% oxybutynin spray and oral oxybutynin), compared to topical placebo. The 10% oxybutynin spray group reported a significant improvement in QoLQ from baseline to day 35. Ten percent oxybutynin spray exhibits systemic effects and should be studied in larger studies to evaluate its role in management of patients with axillary HH.

## Data Availability

The data supporting this study are available upon request to the corresponding author, SN, due to privacy and ethical restrictions.
